# End-to-End Timeliness of Blood Culture Diagnostics: A One-Month Observational Study of 5121 Bottles

**DOI:** 10.3390/antibiotics15040335

**Published:** 2026-03-26

**Authors:** Carlotta Magrì, Damiano Squitieri, Barbara Fiori, Tiziana D’Inzeo, Maurizio Sanguinetti, Brunella Posteraro, Giulia Menchinelli

**Affiliations:** 1 Dipartimento di Scienze Biotecnologiche di Base, Cliniche Intensivologiche e Perioperatorie, Università Cattolica del Sacro Cuore, 00168 Rome, Italy; carlotta.magri@unicatt.it (C.M.); damiano.squitieri@unicatt.it (D.S.); tiziana.dinzeo@unicatt.it (T.D.); brunella.posteraro@unicatt.it (B.P.); 2 Dipartimento di Scienze di Laboratorio ed Ematologiche, Fondazione Policlinico Universitario A. Gemelli IRCCS, 00168 Rome, Italy; barbara.fiori@policlinicogemelli.it (B.F.); giulia.menchinelli@policlinicogemelli.it (G.M.); 3 Unità Operativa “Medicina di Precisione in Microbiologia Clinica”, Direzione Scientifica, Fondazione Policlinico Universitario A. Gemelli IRCCS, 00168 Rome, Italy

**Keywords:** antimicrobial susceptibility testing, blood culture, diagnostic workflow, laboratory staffing, MALDI TOF mass spectrometry, turnaround time

## Abstract

**Background/Objectives**: To quantify end-to-end timeliness of the blood culture (BC) diagnostic workflow over one month using operational key performance indicators (KPIs)—transportation time (TT), time to detection (TTD), time to preliminary report (TTPR), and time to antimicrobial susceptibility testing (AST; TTAST)—and to identify actionable bottlenecks. **Methods**: This retrospective observational analysis included BC bottles processed between 29 September and 29 October 2023 at a large tertiary-care hospital in Italy. KPIs were computed from laboratory information system (LIS) timestamps and structured observations and were summarized as medians (interquartile range [IQR]). **Results**: 44.7% (2290/5121) of bottles reached the laboratory within 2 h (median 2.2 h, IQR 1.3–3.7), suggesting pre-analytical delays. Among adult bottles (*n* = 4995), 68.9% were underfilled (<8 mL), 12.9% met the 8–10 mL target, and 18.2% were overfilled (>10 mL). There were 932 positive bottles (18.2%), with a nocturnal peak in instrument flags despite reduced staffing. Median TTD was 12.6 h (IQR 8.9–18.4), with earlier detection for Gram-negatives than Gram-positives and yeasts (11.9, 14.5, and 30.9 h). In bacterial-positive bottles with complete timestamps (*n* = 294), median TTPR was 3.8 h (IQR 1.7–8.8); median TTAST was 19.2 h (IQR 14.3–27.8). From collection, median times were 17.9 h (IQR 14.2–23.1) to the preliminary report and 36.0 h (IQR 28.8–48.7) to the AST result. **Conclusions**: Within-laboratory steps were generally rapid, whereas transport planning and collection volumes emerged as major bottlenecks. Targeted interventions—enforcing ≤2 h TT and training to achieve an 8–10 mL fill—should further improve BC turnaround time.

## 1. Introduction

Blood culture (BC) remains the cornerstone for diagnosing bloodstream infections and guiding therapy; however, its clinical utility depends not only on analytical accuracy but also hinges on operational timeliness from collection to reporting under routine conditions [1,2]. However, end-to-end performance is rarely quantified at the BC bottle level; most reports examine single workflow segments (e.g., transportation time or time to detection) or rely on patient-level proxies (e.g., time to first antimicrobial/effective therapy), which do not localize operational delays within the laboratory [3,4,5,6]. Although recent technologies—such as automated incubation BC systems, MALDI-TOF mass spectrometry–based identification, and rapid, direct-from-BC antimicrobial susceptibility testing (AST)—have markedly accelerated analytical steps [7,8,9,10,11,12], delays often persist in pre-analytical logistics and in reporting handoffs (handover of results/tasks between staff or shifts), particularly in transport planning, bottle fill volume, report communication, and off-hours staffing, thereby limiting the clinical impact of otherwise fast diagnostic tools [6,13].

To address this gap, we conducted a one-month, bottle-level evaluation of the BC diagnostic workflow at a tertiary-care center using operational key performance indicators aligned with daily practice. We examined pre-analytical performance—transportation time (TT) and adult bottle fill volume—and analytical performance, quantifying time to detection (TTD) for all positive bottles (bacteria and yeasts) and, in accordance with institutional pathways, time to preliminary report (TTPR) and time to AST result (TTAST) for bacterial-positive bottles only (as yeast-positive bottles are managed through a dedicated mycology pathway). Our aim was to characterize real-world distributions, identify operational bottlenecks, and outline practical changes implementable within current standard operating procedures (SOPs). Evaluation of patient-level outcomes was outside the scope of this operational study.

## 2. Results

### 2.1. Cohort and Analysis Sets

Over the one-month period, 5121 BC bottles were received.

**Figure 1:**
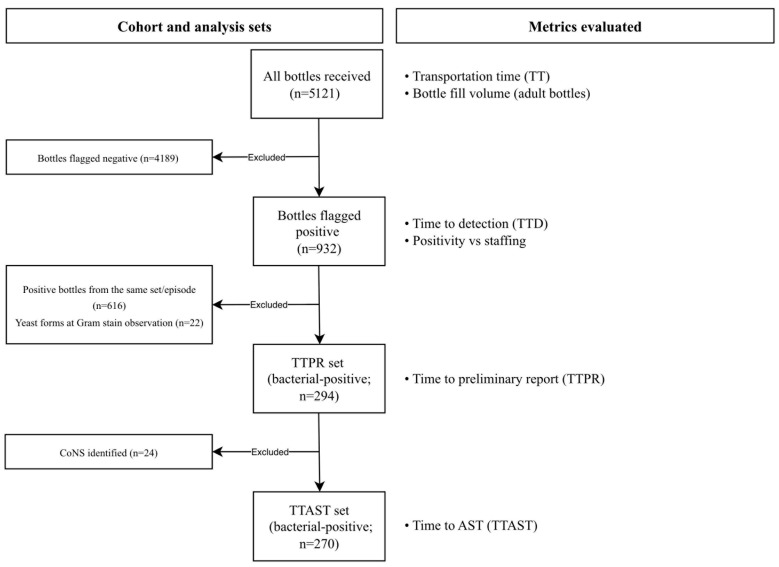
Flowchart of included bottles and analysis sets. One-month cohort of all BC bottles (*n* = 5121). Negative bottles (*n* = 4189) contribute only to pre-analytical metrics (TT and adult bottle fill volume). Among positives (*n* = 932), yeasts at Gram stain observation (*n* = 22) are indicated; all positives enter TTD. Only bacterial-positive bottles contribute to TTPR (after excluding same-set/episode positives; *n* = 294) and to TTAST (after excluding CoNS not processed to AST; *n* = 270). Left-side arrows labeled “Excluded” denote subsets removed from analysis. The right panel lists the metrics evaluated for each set; positivity versus staffing is shown for the positive set (*n* = 932) as a context analysis (not a KPI). Abbreviations: AST, antimicrobial susceptibility testing; CoNS, coagulase-negative staphylococci; TT, transportation time; TTD, time to detection; TTPR, time to preliminary report; TTAST, time to AST.

Figure 1 shows the cohort partitioning and analysis sets. Negative bottles (*n* = 4189) contributed only to pre-analytical metrics (TT and bottle fill volume). The remaining positive bottles (*n* = 932) constituted the TTD set. Within positive bottles—after excluding additional bottles from the same positive set to avoid double counting—*n* = 294 bacterial-positive bottles had complete timestamps for TTPR; among these, *n* = 24 with coagulase-negative staphylococci were not processed to AST in accordance with SOPs, yielding *n* = 270 bottles with complete timestamps for TTAST. Yeast-positive bottles were included in TTD but did not contribute to TTPR/TTAST analyses, per institutional pathways.

All endpoint distributions deviated from normality according to the Anderson–Darling test (*p* < 0.001); therefore, results are summarized as medians with interquartile range (IQR).

### 2.2. Transportation Time (TT) Analyses

**Figure 2:**
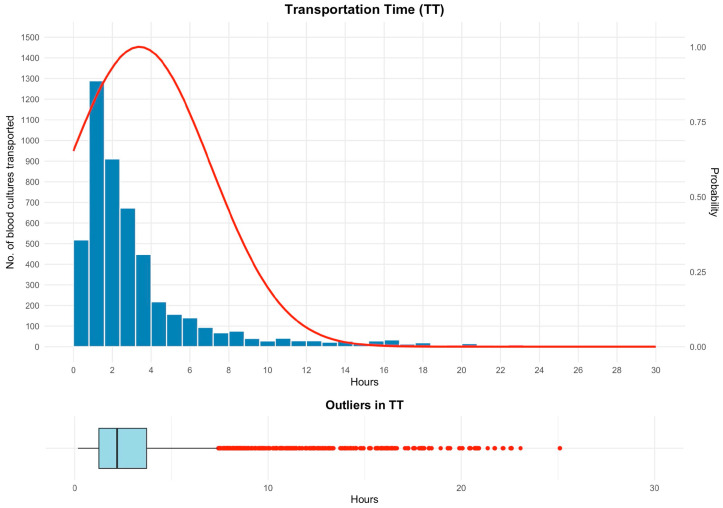
Transportation time (TT). Histogram of time from order (proxy for collection) to laboratory receipt for all bottles (*n* = 5121) with an overlaid smoothed probability density curve (right *y*-axis), showing an early peak and a long right tail. The lower panel shows a boxplot (box = IQR, line = median; whiskers to 1.5 × IQR) with outlier points, highlighting numerous late arrivals. Abbreviations: IQR, interquartile range.

In the full monthly cohort (*n* = 5121), 44.7% of bottles (*n* = 2290) arrived at the laboratory within 2 h (Figure 2). TT was right-skewed (range, 0.17–25.1 h; median, 2.2 h; IQR, 1.3–3.7 h). Rare but extreme late arrivals formed a long tail. Receipt volume showed a morning spike around 08:00–10:00, consistent with batching at shift change; overnight flow was lower but continuous, suggesting opportunities to smooth dispatch schedules across the night-to-day transition.

### 2.3. Bottle Fill Volume (Adult Bottles) Analyses

**Figure 3:**
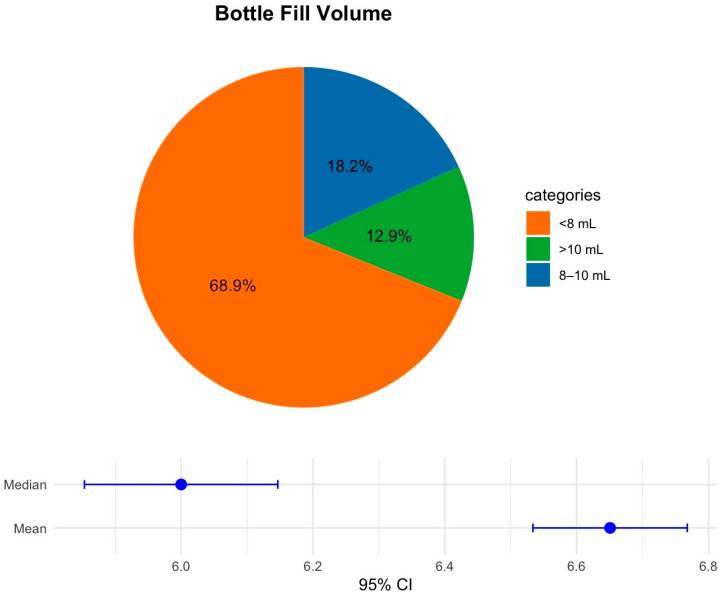
Bottle fill volume (adult bottles). Pie chart of adult BC bottles (*n* = 4995) categorized as underfilled (<8 mL), within the manufacturer-recommended target (8–10 mL), or overfilled (>10 mL); slice labels report percentages. The lower panel displays the median and mean volumes (points) with 95% confidence intervals (error bars). Abbreviations: BC, blood culture.

Among adult bottles (*n* = 4995), underfilling (<8 mL) predominated (68.9%); target fills (8–10 mL) accounted for 12.9%, and overfilling (>10 mL) for 18.2% (Figure 3). The median volume was 6.0 mL (95% CI, 5.9–6.2 mL) and the mean volume was 6.7 mL (95% CI, 6.5–6.8 mL). These distributions support targeted training and feedback to improve collection technique and documentation.

### 2.4. Time to Detection (TTD) Analyses

**Figure 4:**
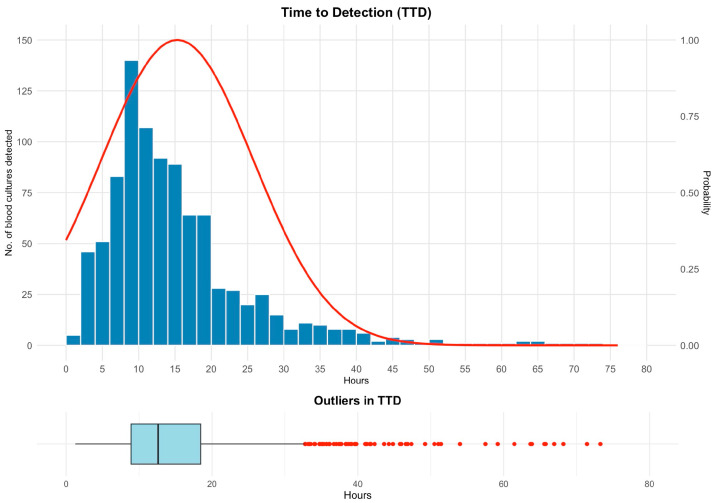
Time to detection (TTD). Histogram of time from incubation start to the instrument-positive flag for all positive bottles (*n* = 932), with an overlaid smoothed probability density curve (right *y*-axis), showing an early detection peak and a long right tail. The lower panel shows a boxplot (box = IQR, line = median; whiskers to 1.5 × IQR) with outlier points, highlighting prolonged detection times. Abbreviations: IQR, interquartile range.

Across positive bottles (*n* = 932), TTD displayed a right-tailed distribution (median 12.6 h, IQR 8.9–18.4 h; maximum 73.2 h) (Figure 4). Most detections occurred early: *n* = 677 (72.6%) ≤18 h and *n* = 796 (85.4%) ≤24 h. In monomicrobial bottles (*n* = 834; after excluding 98 polymicrobial bottles), Gram-negative bacteria dominated early detection windows (1–10 h: 63.6%) compared with Gram-positive bacteria (36.4%) and yeasts (0.0%), whereas Gram-positive bacteria and yeasts predominated in later windows (>40 h: 68.0% and 32.0%, respectively; no Gram-negative bacteria detected).

### 2.5. Positivity Patterns and Staffing Analyses

**Figure 5:**
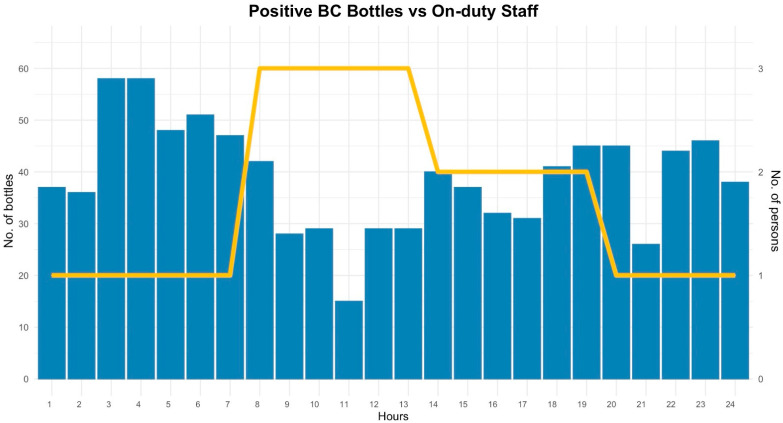
Positivity versus staffing. Hourly counts of instrument positive flags over the one-month window (bars, left *y*-axis), overlaid with on-duty staff assigned to the BC workflow (line, right *y*-axis). Staffing follows the typical roster—3 operators 08:00–14:00, 2 operators 14:00–20:00, and 1 operator 20:00–08:00—highlighting nocturnal peaks during periods of minimum staffing. Abbreviations: BC, blood culture.

The overlay of positive flags and on-duty staffing (Figure 5) shows nocturnal peaks in instrument positive flags when staffing is at its minimum (one operator, 20:00–08:00), compared with two to three operators during the morning (08:00–14:00) and evening (14:00–20:00) shifts. By shift, positive bottles were 170 (08:00–14:00), 231 (14:00–20:00), and 531 (20:00–08:00), reinforcing the nighttime surge in actionable workload.

### 2.6. Time to Preliminary Report (TTPR) and Time to AST (TTAST) Analyses

**Figure 6:**
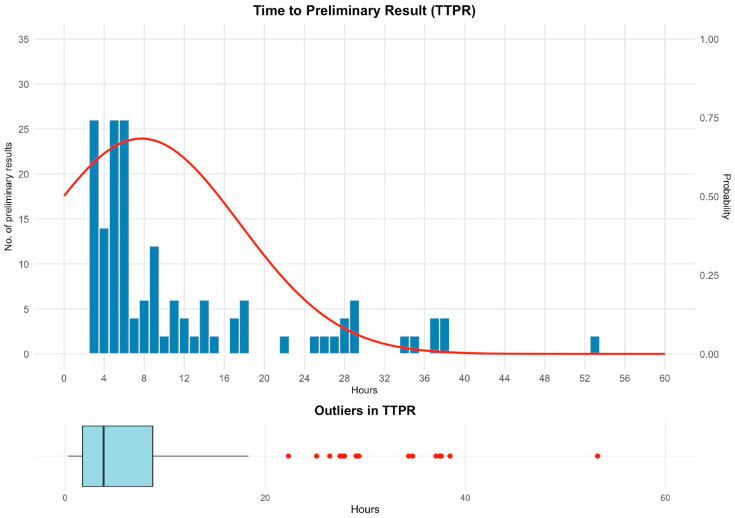
Time to preliminary report (TTPR). Histogram of time from the instrument-positive flag to the first clinician-actionable preliminary report (phone call) for bacterial-positive bottles with complete timestamps (*n* = 294), with an overlaid smoothed probability density curve (right *y*-axis), showing an early peak and a long right tail. The lower panel shows a boxplot (box = IQR, line = median; whiskers to 1.5 × IQR) with outlier points. Abbreviations: IQR, interquartile range.

For bacterial-positive bottles with complete preliminary report timestamps (*n* = 294), the median TTPR was 3.8 h (IQR, 1.7–8.8 h), with a small number of delays >20 h (Figure 6). By shift, the median TTPR was 2.9 h (IQR, 1.6–9.3 h) for 08:00–14:00, 2.1 h (IQR, 1.8–3.8 h) for 14:00–20:00, and 2.2 h (IQR, 1.8–8.8 h) for 20:00–08:00.

**Figure 7:**
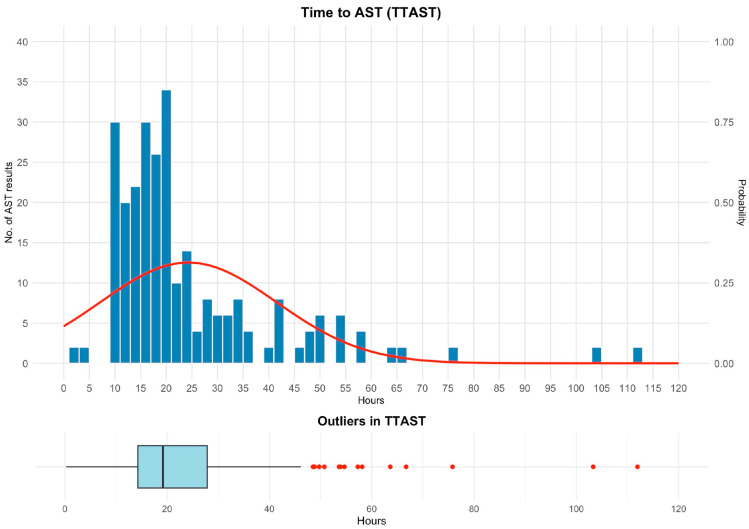
Time to AST (TTAST). Histogram of time from the instrument-positive flag to AST result release in the LIS for bacterial-positive bottles with AST results (*n* = 270), with an overlaid smoothed probability density curve (right *y*-axis), showing an early peak and a long right tail. The lower panel shows a boxplot (box = IQR, line = median; whiskers to 1.5 × IQR) with outlier points. Abbreviations: AST, antimicrobial susceptibility testing; IQR, interquartile range; LIS, laboratory information system.

For bacterial-positive bottles with AST results released (*n* = 270), the median TTAST was 19.2 h (IQR, 14.3–27.8 h), with most results issued within 12–24 h (Figure 7). Outliers >30 h mainly reflected polymicrobial bottles or failed direct identification requiring prior colony growth on solid medium.

### 2.7. Collection-to-Report and Collection-to-AST Analyses

From BC collection, the median time to the first clinician-actionable preliminary report was 17.9 h (IQR 14.2–23.1), and the median time to the AST result was 36.0 h (IQR 28.8–48.7).

## 3. Discussion

In this one-month bottle-level evaluation of the BC workflow, we quantified operational timeliness and localized where delays arise under routine conditions. Pre-analytically, only 44.7% of bottles arrived within 2 h, with a long right tail indicating variability up to laboratory receipt. Among positives (*n* = 932), TTD was heavy-tailed (median, 12.6 h), with earlier detection for Gram-negatives and later for Gram-positives/yeasts. Within the laboratory, actionability was generally prompt (TTPR median 3.8 h; TTAST median 19.2 h), though it was prolonged for polymicrobial bottles or when direct ID was not feasible. From collection, the median times were 17.9 h to the preliminary report and 36.0 h to the AST result, integrating pre-analytical and post-flag steps.

When discussing prior studies, we use the umbrella term turnaround time (TAT) as defined by each author; our stepwise KPIs (TT, TTD, TTPR, TTAST) are reported as per Methods. Our findings align with earlier work showing that pre-analytical organization strongly shapes overall TAT. Lamy et al. [2] advocate monitoring ≤2 h transport and enforcing adult fill-volume targets of 8–10 mL, leveraging automated volume estimation on VIRTUO. Our bottle-level, stepwise distributions complement studies centered on aggregate time-to-result or rate-based indicators. In Italian hospitals, Palmieri et al. [4] evaluated the BD DREAM™ digital remote KPI tool across ≈235,000 bottles, documenting mean per-bottle volumes around 6.5 mL—below the recommended 8–10 mL [14]—with the lowest mean (≈5.75 mL) in medical wards; these data support systematic monitoring and feedback on fill volume. Consistently, a prior survey reported that only a minority of laboratories routinely monitored under- or over-inoculation [15].

Multicenter audits show substantial end-to-end delays. In Israeli acute-care hospitals, Temkin et al. [16] reported a median BC processing TAT ≈51.2 h (IQR 33.9–78.0), partitioned into ≈4.4 h pre-analytical (1.7–12.5), ≈15.9 h incubation-to-detection (10.2–23.6), ≈4.5 h detection-to-Gram (1.5–10.7), and ≈30.9 h detection-to-identification (22.0–41.9), plus ≈8.6 h attributable to off-hours operations—evidence that organization and staffing patterns materially affect timeliness. In Chinese teaching hospitals, Liu et al. [17] reported collection-to-AST TAT medians ≈2.7 days for bacteria and ≈3.7 days for yeasts, with early-morning transport lags and overnight slowdowns in loading/reporting; notably, moving from a 24/7 central-lab model to delayed loading/processing was associated with longer collection-to-AST TAT (increasing from ≈2.35 to ≈3.25 days). The authors further argued that pathway design (inoculum, organism type, “fast-track” rules, equipment, rapid AST) materially influences TAT [17]. Before–after evidence shows that reorganizing services can compress TAT: Paluch et al. [18] observed significant reductions in clinically significant BC TAT after a 24/7 reorganization with continuous loading/automation.

Against this backdrop, our stepwise, bottle-level analysis—combining distributions for TT, TTD, TTPR, and TTAST with an explicit overlay of positivity timestamps and staffing—adds granularity. We confirm the nocturnal peak in instrument-positive flags yet find overnight TTPR medians comparable (and at times shorter), plausibly because night workload is concentrated on BC tasks, whereas daytime benches must absorb other urgent streams (e.g., WASP loading and MALDI-TOF). No clear delays in reporting were observed during night shifts, and time to incubation was not directly assessed in this study. This pattern underscores that technology alone (e.g., direct MALDI-TOF and direct AST) is insufficient: meaningful gains require concurrent attention to pre-analytical logistics (dispatch/batching, fill volume) and within-lab handoffs (smear, preliminary authorization, and AST setup), as highlighted by prior reports.

Four operational areas emerge as practical targets. First, transport and handoffs: smoothing early-morning dispatches and using an “in-transit” dashboard should increase ≤2 h arrivals and compress the right tail. Potential strategies to mitigate night-time workflow challenges include optimization of staffing models, implementation of dedicated or rapid transport systems for BC bottles, and increased reliance on automation to minimize delays in incubation and processing. Second, collection quality issues showed that underfilling was frequent. Notably, these findings were observed despite the availability of an institutional BC protocol and standardized checklists, suggesting that variability in real-world implementation and contextual factors may impact adherence to recommended collection volumes. Continuous feedback leveraging VIRTUO volume estimation and focused coaching should improve adherence to the 8–10 mL target. In this context, structured and periodically reinforced training programs, combined with real-time feedback, may help standardize practices and reduce variability in collection performance. Third, post-flag flow: maintain rapid issuance of clinician-actionable preliminaries (Gram/direct MALDI-TOF when feasible; BCID2 when polymicrobial or when direct ID is not feasible) and reduce the small cluster of >20 h outliers by (i) escalation paging, (ii) standardized fast-track preliminary templates for common Gram-negative morphologies, and (iii) prioritizing morning setup for overnight positives. When direct ID or AST is inconclusive, trigger predefined confirmatory steps (e.g., broth microdilution or targeted resistance assays) to finalize results without rework. Fourth, physical layout matters: co-locating accessioning, VIRTUO, Gram/microscopy, MALDI-TOF, and VITEK 2 reduces walking distance and handoffs, helping to shorten TTPR and TTAST during peak hours.

Strengths include an end-to-end, bottle-level perspective, minute-level timestamps, and inclusion of polymicrobial/fastidious cases for real-world relevance. Limitations include the single-center, one-month horizon; absence of patient-level outcomes; and residual risk of timestamp misclassification despite reconciliation. The analysis is descriptive by design: we report distributions rather than summing medians across segments, which would be inappropriate for conditional, right-skewed intervals. Another limitation is that TT was computed as the interval from order to laboratory receipt because the laboratory information system (LIS) equates “collection” with order entry and does not store a separate venipuncture time. When order precedes venipuncture, TT may overestimate transport/dispatch by including ward-side scheduling; when they coincide, TT reflects dispatch/transport delays (e.g., batching before pickup). Accordingly, TT should be interpreted as the pre-analytical pathway up to laboratory receipt rather than a pure transport measure. We could not disaggregate ward-side scheduling from transport in this dataset. A further limitation of this study is that blood volume measurements were available only for adult BC bottles, precluding separate analysis of pediatric samples, which may be particularly sensitive to underfilling and low bacterial load. In addition, clinical indications for blood culture collection were not systematically assessed, as patient-level clinical data were not included in this analysis; therefore, interpretation of negative results in relation to clinical context falls outside the scope of this study.

Finally, expert viewpoints have long argued that BC optimization is pathway-specific and should precede or accompany the adoption of rapid methods. Banerjee et al. [19] emphasized tailoring BC practices to local resistance patterns, antibiotic use, patient mix, staffing, and laboratory setup, with continuous monitoring and feedback to ensure quality before adopting new technologies. Ten years on, our experience suggests that analytical steps can be made consistently rapid under direct methods, whereas sustained gains now depend on tightening pre-analytical logistics and standardizing within-lab handoffs. In this context, our diagnostic workflow is broadly consistent with international recommendations from the Clinical and Laboratory Standards Institute (CLSI) and the European Committee on Antimicrobial Susceptibility Testing (EUCAST), while the implementation of direct identification and AST approaches represents an adaptation aimed at improving turnaround times; however, comparison with established quality benchmarks highlights pre-analytical factors—particularly transport-related time and blood volume adequacy—as key areas for improvement.

## 4. Materials and Methods

### 4.1. Study Design and Setting

We conducted a retrospective observational evaluation of the blood culture (BC) diagnostic workflow at the clinical microbiology laboratory of the Fondazione Policlinico Universitario “A. Gemelli” IRCCS (Rome, Italy), a tertiary-care hospital. The observation window covered a contiguous one-month period (29 September–29 October 2023). Analyses were performed at the BC bottle level on de-identified laboratory records under routine operations; no patient-level outcomes were collected.

During the study period, an institutional BC collection protocol was in place, supported by standardized checklists for peripheral venipuncture, central venous catheter, and implantable port blood sampling. However, adherence to these procedures was not directly assessed.

### 4.2. Laboratory Organization and Logistics

The clinical microbiology laboratory operates 24/7 under a shift system. BC bottles are received continuously, either by hand delivery or via the hospital pneumatic tube system, depending on the ward. Dispatches are ad hoc rather than scheduled, and bottles arrive individually or in small batches. Upon receipt, bottles are registered and loaded onto the BC instrument (as specified below) without delay. BC-dedicated staffing typically consists of three operators (08:00–14:00), two (14:00–20:00), and one overnight (20:00–08:00). Instrument-positive flags are automatically recorded in the laboratory information system (LIS) via middleware; preliminary reports are communicated by phone call according to standard operating procedures (SOPs), whereas AST results are released in the LIS after authorization.

### 4.3. Instruments and Laboratory Procedures

Bottles (BacT/ALERT^®^ FA Plus/FN Plus; bioMérieux, Marcy-l’Étoile, France) were incubated and continuously monitored on the BacT/ALERT^®^ VIRTUO^®^ system (bioMérieux), integrated with the hospital LIS via MYLA^®^ middleware (bioMérieux). At loading, VIRTUO recorded bottle fill volume and generated instrument-positive flags. According to the laboratory’s SOPs, positive bottles underwent: (i) Gram staining—assessing mono-/polymicrobial morphology and the presence of yeasts; (ii) subculture on solid media via the WASP^®^ Walk-Away Specimen Processor (COPAN Italia S.p.A., Brescia, Italy) plate inoculation (streaking); (iii) if monomicrobial, direct MALDI-TOF mass spectrometry identification (ID) using the MALDI Biotyper system (Bruker Daltonics, Bremen, Germany); and (iv) direct AST using the VITEK^®^ 2 system (bioMérieux) [20]. For polymicrobial positive BCs—or when direct ID from the positive bottle was not feasible—a syndromic molecular panel (BioFire^®^ FilmArray^®^ BCID2; bioMérieux) was used to issue the first clinician-actionable preliminary report by phone call.

When appropriate, AST was also performed on isolates recovered from subculture. Additionally, targeted rapid resistance assays were applied when indicated: eazyplex^®^ MRSA Plus, eazyplex^®^ VanA/VanB (AmplexDiagnostics GmbH, Gars am Inn, Germany), and NG-Test^®^ CARBA 5/NG-Test^®^ CTX-M MULTI (NG Biotech, Guipry-Messac, France). In some cases, reflex AST (e.g., cefiderocol disk diffusion) was performed to confirm AST results prior to final reporting.

**Figure 8:**
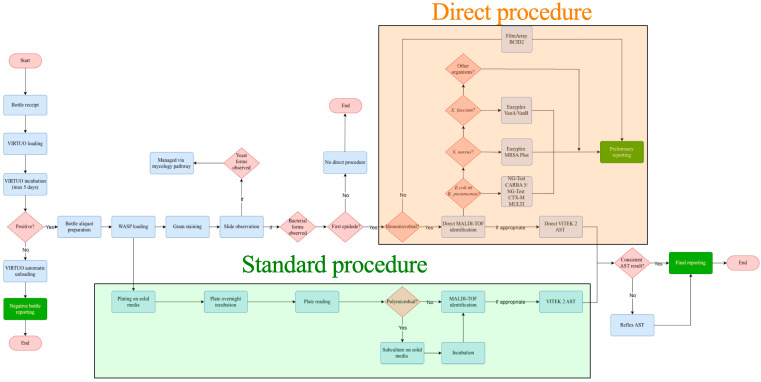
Workflow comparison between standard and direct blood culture processing. The diagram illustrates the sequence of steps from bottle receipt to preliminary reporting and final release in the laboratory information system (LIS). The upper (red-shaded) section represents the direct procedure, while the lower (green-shaded) section shows the standard procedure. Rectangles denote process steps, including VIRTUO loading and incubation (up to five days), Gram staining, WASP plating and subculture, MALDI Biotyper identification, FilmArray BCID2 testing, and VITEK 2 antimicrobial susceptibility testing (AST), with reflex AST when required. Diamonds indicate representative decision nodes (e.g., positive signal, presence of yeast forms, first episode, monomicrobial growth, and consistency of AST results). The direct procedure applies only to bottles from the first (index) episode, defined according to previously published criteria [21]. Abbreviations: AST, antimicrobial susceptibility testing; BC, blood culture; LIS, laboratory information system; WASP, Walk-Away Specimen Processor.

Figure 8 provides a schematic representation of the BC diagnostic workflow, highlighting the two procedures. The direct procedure (Gram stain; direct MALDI-TOF identification when feasible; BCID2 when polymicrobial or when direct ID was not feasible; and direct VITEK 2 AST, with or without targeted resistance-gene detection) was applied only to bacterial-positive bottles from the first (index) episode. The index status was assigned per patient during routine operations according to our previously published criteria [21]. Bottles not meeting these criteria—including non-index positives within the same episode—were managed through the standard procedure (subculture on solid media via WASP plate inoculation, with 24 h reading; MALDI-TOF and AST from colonies). Bottles with yeast forms on Gram stain were processed via the mycology pathway.

### 4.4. Operational Key Performance Indicators Evaluated

Timeliness was assessed using four prespecified operational key performance indicators (KPIs), reported in hours (h): transportation time (TT), time to detection (TTD), time to preliminary report (TTPR), and time to AST result (TTAST). TT was defined as the interval from collection to laboratory receipt; TTD, from incubation start to the instrument positive flag; TTPR, from the instrument positive flag to the first clinician-actionable preliminary report by phone call; and TTAST, from the instrument positive flag to AST result release in the LIS. In our LIS, the “collection” timestamp coincides with order entry (venipuncture time is not recorded separately); therefore, TT should be interpreted as the pre-analytical interval from order/collection to receipt, rather than a pure transport time. KPI definitions followed the laboratory’s SOPs and were applied uniformly across the observation window. For TT, we used a ≤2 h target for descriptive compliance [2,14]. For context, we also summarized two composite intervals: time from blood culture collection to the first clinician-actionable preliminary report, and time from blood culture collection to AST result release. These intervals were computed directly from timestamps and reported descriptively; they were not used as primary KPIs.

### 4.5. Data Sources and Variables

We combined LIS/middleware timestamps and metadata with structured manual observations by trained personnel, recorded via a standardized checklist to capture steps not routinely logged (e.g., start of Gram staining and microscopic examination of the stained smear; direct identification by MALDI-TOF or BCID2 when attempted; and preliminary reporting). Times were noted to the minute using clocks synchronized daily with LIS terminals and linked to records via barcodes, then reconciled with LIS data during cleaning. Extracted time-stamped fields included: order/collection time (order entry, used as a proxy for venipuncture); laboratory receipt time; incubation start time; instrument positive-flag time; preliminary report time (first clinician-actionable phone call); identification release time (if applicable); and AST release time (if applicable). Operational covariates included shift (morning 08:00–14:00; evening 14:00–20:00; night 20:00–08:00). Bottle fill volume was recorded against the VIRTUO manufacturer-recommended 8–10 mL target. Adult bottles were operationally defined via the LIS/middleware bottle-type field (BacT/ALERT FA Plus and FN Plus); non-adult bottle types (e.g., BacT/ALERT PF Plus) were excluded from the fill-volume analysis because their volumes were not monitored. Records contained no direct identifiers; bottles were tracked via barcodes and de-identified internal IDs.

### 4.6. Inclusion and Exclusion Criteria

All BC bottles received during the observation month were eligible. Prespecified exclusions were: (a) bottles canceled before incubation; (b) invalid requests at laboratory receipt; (c) test/validation bottles; (d) duplicate records generated during downtime recovery; and (e) records with irreconcilable missing critical timestamps after data reconciliation. TTD analyses were limited to positive bottles (bacteria and yeasts). TTPR and TTAST analyses were restricted to bacterial-positive bottles with the corresponding complete timestamps. Polymicrobial and fastidious cases were included; these bottles were annotated as polymicrobial or fastidious in the dataset. Bottles showing yeast forms on Gram stain were managed through the mycology pathway and were not part of TTPR/TTAST.

### 4.7. Statistical Analysis

Continuous KPIs were summarized as median (interquartile range, IQR); means (standard deviation, SD) were reported where informative. Distributions were assessed visually using histograms with overlaid smoothed probability density curves and box plots, and with the Anderson–Darling test given the expected right-skew and heavy tails. Stratified descriptive summaries were produced by shift; no prespecified hypothesis testing was performed. All time intervals were computed from minute-level LIS timestamps and expressed in hours (h). Analyses were conducted in R software (version 2023.09.1).

## 5. Conclusions

Focused, low-complexity actions—enforcing ≤2 h pre-analytical pathways, improving fill volume, and tightening post-flag workflows—should further improve overall TAT in BC diagnostics. Prospective monitoring over several months, ideally using statistical process control charts, can verify sustained gains and reduce variability. This can be operationalized via LIS/middleware dashboards or commercial analytics platforms (e.g., CLARION™ and bioMérieux), enabling automated data pulls, shift-level drilldowns, and alerts when KPIs breach predefined thresholds.

## Data Availability

Deidentified summary data and analysis code are available from the corresponding author upon reasonable request and subject to institutional approvals. Datasets containing potentially identifying operational timestamps cannot be shared publicly to protect institutional confidentiality.
